# A Nonorthogonal Configuration Interaction Approach to Singlet Fission in Perylenediimide Compounds

**DOI:** 10.1021/acs.jpca.3c04975

**Published:** 2023-11-15

**Authors:** C. Sousa, A. Sánchez-Mansilla, R. Broer, T. P. Straatsma, C. de Graaf

**Affiliations:** † Departament de Ciència de Materials i Química Física and Institut de Química Teòrica i Computacional, Universitat de Barcelona, 08028 Barcelona, Spain; ‡ Departament de Química Física i Inorgànica, Universitat Rovira i Virgili, 43007 Tarragona, Spain; ¶ Zernike Institute of Advanced Materials, University of Groningen, 9747 AG Groningen, The Netherlands; § National Center for Computational Sciences, Oak Ridge National Laboratory, Oak Ridge, Tennessee 37831-6373, United States; ∥ Department of Chemistry and Biochemistry, University of Alabama, Tuscaloosa, Alabama 35487-0336, United States; ⊥ ICREA, Pg. Lluís Companys 23, 08010 Barcelona, Spain

## Abstract

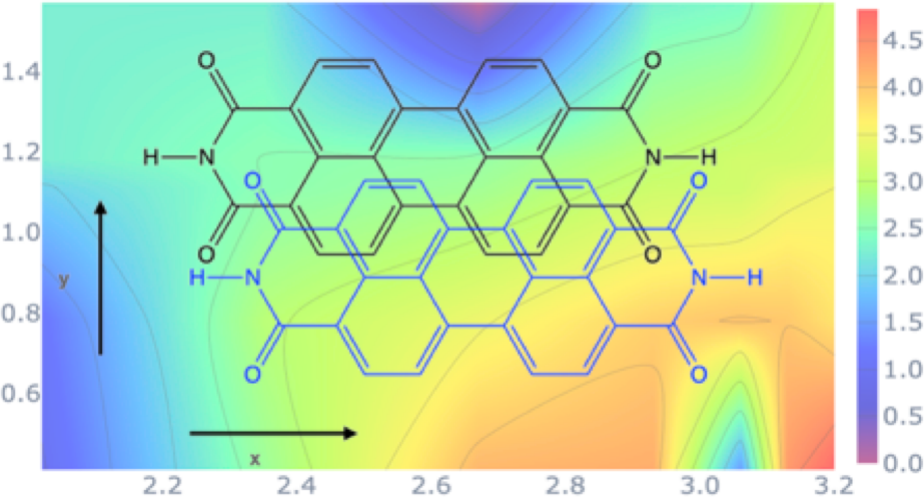

Perylenediimide molecules constitute a family of chromophores that undergo singlet fission, a process in which an excited singlet state converts into lower energy triplets on two neighboring molecules, potentially increasing the efficiency of organic solar cells. Here, the nonorthogonal configuration interaction method is applied to study the effect of the different crystal packing of various perylenediimide derivatives on the relative energies of the singlet and triplet states, the intermolecular electronic couplings, and the relative rates for singlet fission. The analysis of the wave functions and electronic couplings reveals that charge transfer states play an important role in the singlet fission mechanism. Dimer conformations where the PDI molecules are at large displacements along the long axis and short on the short axis are posed as the most favorable for singlet fission. The role of the substituent at the imide group has been inspected concluding that, although it has no effect in the energies, for some conformations it significantly influences the electronic couplings, and therefore, replacing this substituent with hydrogen may introduce artifacts in the computational modeling of the PDI molecules.

## 1 Introduction

Singlet fission (SF), a multiple exciton generation process occurring in organic molecules, turns out to be a promising process to increase the conversion efficiency of photovoltaic devices.^1,2^ SF takes place in organic chromophores in which the excitation energy of the lowest singlet excited state is approximately twice the excitation energy of the lowest triplet, *E*(*S*_1_) ≳ 2*E*(*T*_1_) .^3,4^ Hence, SF is thermodynamically favorable when the energy difference *E*(*S*_1_) – 2*E*(*T*_1_) is positive and small or slightly negative (by less than ∼0.2 eV), i.e., for slightly exothermic or isoenergetic processes. Large exothermicity induces heat energy losses, which results in a decrease of SF efficiency. Moreover, to avoid possible triplet–triplet deactivation channels, the excited triplet and quintet states should lie at least two times higher in energy than the low-lying triplet state, *E*(*T*_2_/*Q*_1_) > 2*E*(*T*_1_). Optimal chromophore molecules require good light-harvesting properties, with intense absorption bands in the visible spectra, and be chemical stable.^5^ For these reasons, π-conjugated organic molecules are potentially suitable SF candidates.

The singlet fission process starts by absorption of light generating a singlet excited state in a molecule, *S*_1_, that combines with a neighboring molecule in the ground state, *S*_0_, producing two triplet states *T*_1_, one on each of the two molecules, overall coupled to singlet spin, ^1^*TT*. This process is radiationless and spin-allowed and hence, is expected to be fast, usually in the picosecond or subpicosecond time scale, thus competing with vibrational relaxations.^6,7^ In the next step, the resulting singlet state ^1^*TT* can be separated into two decoupled triplet states, thus doubling the number of electron–hole pairs in the system. Despite the fact that SF usually takes place between neighboring molecules in a molecular crystal, it can also occur on a single molecule, so-called intramolecular singlet fission.^8,9^

Although SF is a complex, multistep process, and is not yet fully understood, the basic mechanism has been rationalized in terms of a direct or indirect mechanism.^3^ The direct mechanism involves a straight two-electron process from a *S*_0_*S*_1_ state, accessed by light absorption, to a ^1^*TT* state, while in the indirect mechanism, the process is mediated by charge transfer states between neighboring chromophores. The involvement of charge transfer states can be envisaged either as virtual states facilitating the ^1^*TT* formation via a superexchange mechanism or as real intermediate states in a sequential two one-electron transfer step process.^7^

Since SF implies energy transfer between neighboring chromophores, the process depends not only on the nature of the particular chromophore but also on the relative orientation of the monomers in the molecular crystal.^10,11^ The energetic condition is that the SF process should be slightly exoergic or isoergic. However, this is not the only parameter that controls the SF rate and yield; intermolecular electronic couplings between neighboring molecules also play a key role in the process. In fact, electronic couplings, which allow for nonadiabatic transitions between different states, are markedly dependent on the particular arrangement of the chromophores.^12^

Singlet fission has been observed in different organic materials showing a slightly exothermic or isoenergetic process, such as pentacene,^13−16^ 1,3-diphenylisobenzofuran (DPIBF),^17,18^ carotenoids,^19^ and their derivatives, but also in materials as tetracene,^20,21^ rubrene,^22,23^ perylene,^24,25^ and related derivatives, where the process is slightly endothermic.

**Figure 1:**
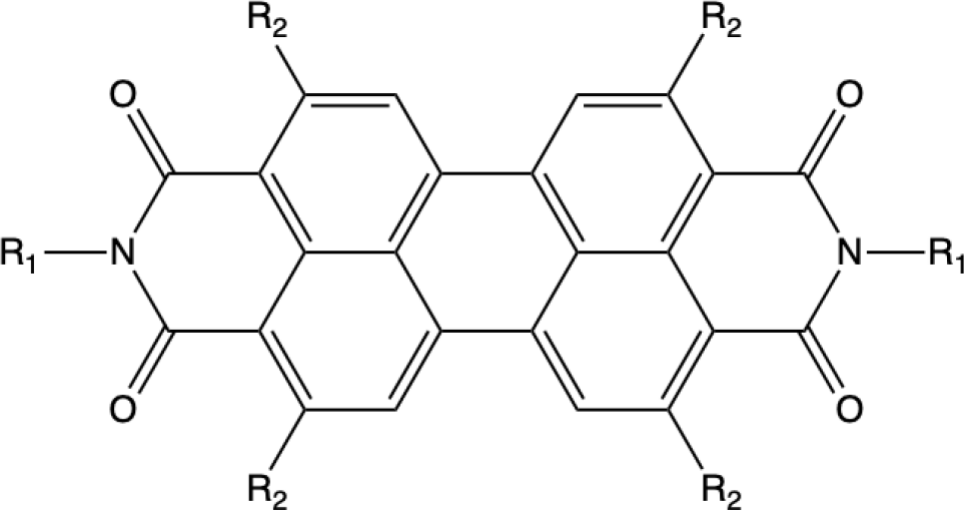
Perylenediimide monomer.

**Figure 2:**
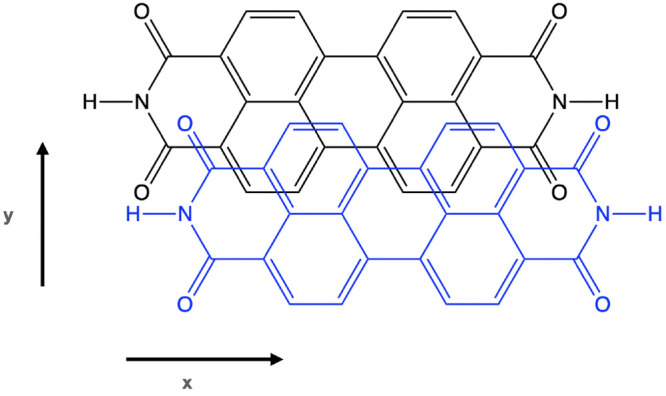
PDI dimer displaying the displacement along the long (*x*) and short (*y*) axes.

Perylene-3,4:9,10-bis(dicarboximide) (PDI) molecule (Figure 1) and its derivatives are chromophores that form ordered π-stacked structures where the crystal packing depends on the different functional groups at the N atom of the imide groups (*R*_1_). They constitute a vast family of versatile dyes for photophysical applications due to their good light-harvesting properties, showing intense absorptions in the visible spectra and thermal and photochemical stability. Moreover, PDI derivatives are suitable chromophores for singlet fission since the energy of the singlet excited state is approximately the sum of the energy of two triplets, fulfilling the thermodynamic condition.^25,26^ These characteristics make PDI derivatives ideal systems to explore the optical properties by changing the substituents at the imide groups and, consequently, the crystal packing. This opens the possibility of modifying the structure to tune their photophysical properties at will, eventually targeting the control and increase of the SF efficiency. In fact, the effect of crystal packing in the SF rates and yields has been experimentally studied by Le et al.^25,27^ by transient absorption and time-resolved photoemission spectroscopy in six different PDI derivatives, showing that triplet excitons can be produced at the picosecond time scale with a maximum yield of 178% when the PDI molecules are arranged cofacially with a slip displacement along the long *x*-axis of ∼3.0 Å and minimal short *y*-axis displacement (see Figure 2 for the definition of the axes). However, the singlet fission created triplets are found to decay to the ground state over tens of nanoseconds. In the theoretical studies of the electronic couplings and SF rates^28^ based on the restricted active space configuration interaction with double spin-flip method,^29^ it has been postulated that displacements ≥2.5 Å along both axis are more favorable to prompt singlet fission. On the other hand, Mirjani et al.^30^ suggested a zero displacement in the short axis and a ∼2.8 Å slip in the long direction as the optimal region for SF. Moreover, the process takes place through a charge transfer enhanced pathway where the charge transfer states act as virtual intermediate states.

In the present work, we study a series of PDI aggregates to analyze the dependence of the two key parameters for singlet fission, SF energy, and intermolecular electronic coupling, on the stacking structure of these molecules in the crystal. From their magnitudes, relative rates are estimated by a simple kinetic model.^28^ This allows one to find dimer arrangements that are optimal for SF, ultimately helping the design of new advantageous materials. To do so, we employ a nonorthogonal configuration interaction approach,^31,32^ based on monomer multiconfigurational wave functions, that fully accounts for orbital relaxation and nondynamical electron correlation. The remaining, mostly dynamical, electron correlation, can be included by a second-order perturbation theory energy correction.^33^ This methodology has the advantage of yielding very short wave functions expressed in terms of many-electron basis functions with a clear diabatic character that can be directly assigned to specific electronic states such as local excitations or charge transfer states. This vastly facilitates the physical interpretation and analysis of the process under study. Moreover, it allows quantification of the effect of the dynamical electron correlation in the properties of interest. Compared to other theoretical approaches previously applied in the computation of electronic couplings, such as simple models considering only the highest occupied and lowest unoccupied orbitals of the interacting molecules^3,34,35^ and density functional theory (DFT) based methods,^36−38^ the present approach is based on *ab initio* multiconfigurational wave functions, which allows researchers to treat all excited states on the same footing and is free of any dependence on the particular choice of the functional.

The paper is organized as follows: the computational approach employed to evaluate the energies and electronic couplings of the PDI derivatives studied is presented in Section 2. Results of the relative energies and character of the relevant electronic states for the PDI monomer and the studied dimers are presented in Section 3, followed by the variation of the energies, electronic couplings, and relative rates with the slip displacement between PDI monomers, from which the optimal geometries for SF can be foreseen. Finally, the effect of the substituent of the imide group on the energies and electronic couplings has been analyzed.

## 2 Computational Approach

The geometry of the ground state, *S*_0_, of the PDI monomer with the *R*_1_ and *R*_2_ substituents modeled by H atoms (see Figure 1), has been optimized by Density Functional Theory (DFT) using different exchange-correlation functionals and the D3 van der Waals dispersion energy correction by Grimme et al.^39^ The functionals applied include two GGA functionals, PBE-D3^40^ and BP86-D3,^41,42^ two hybrid functionals, B3LYP-D3^43,44^ and PBE0-D3,^45^ and two range-separated hybrid functionals, ωB97X-D3^46^ and CAM-B3LYP.^47^ Gaussian type basis sets of triple-ζ plus polarization quality^48^ (def2-TZVP) were applied for all atoms. All DFT calculations were performed with the Orca 4.2.0 code.^49^ In all cases, the optimized geometry of the PDI molecule for the *S*_0_ ground state is planar and has a *D*_2*h*_ symmetry. Differences in interatomic distances and angles among the different functionals are small and do not significantly affect the molecular geometry. The largest internuclear distances are found for the GGA functionals, which differ at most by 0.02 Å compared to the ones obtained applying hybrid functionals, while the angles vary by less than 0.5 degrees. The vertical energies of the lowest singlet and triplet states, *S*_1_ and *T*_1_, have been computed by Time Dependent DFT (TD-DFT) for each optimized structure (Table S1 of the Supporting Information). Results show dispersion of the excitation energies varying the functional, with all values being blue-shifted compared to the available experimental data in solution. The *E*(*S*_1_) – 2*E*(*T*_1_) energy difference is negative in all cases, meaning that the thermodynamic criterion for SF is not satisfied since the process is endoergic by more than 0.2 eV. The structures of the PDI monomer with three different substituents at the *R*_1_ position, a methyl (C1), an ethyl (C2) and an ethylphenyl (EP) group, have been optimized applying the BP86-D3 functional and the same basis set as for the model PDI with *R*_1_ = H.

At the BP86-D3 optimized geometry, state specific Complete Active Space Self Consistent Field (CASSCF) and CAS second-order perturbation theory (CASPT2) calculations on the monomer in vacuum were performed with OpenMolcas.^50,51^ An active space containing 8 π orbitals and 8 electrons has been considered for the PDI monomer. ANO-RCC basis sets^52^ with three different contraction schemes were applied. First, Basis 1 consists of a (5s,4p,2d,1f) contraction for C, N and O atoms and (3s,2p,1d) for H. Basis 2 is composed of a (4s,3p,1d) contraction for C, N and O atoms and (3s,1p) for H. Finally, in Basis 3 a smaller (2s) contraction for the H atoms is applied, while for the rest of the atoms a (4s,3p,1d) contraction is maintained. All electrons were included in the perturbational treatment of the dynamic correlation except the deep-core electrons (1s^2^ for C, N and O). The standard CASPT2 zeroth-order Hamiltonian with ionization potential-electron affinity (IPEA) shift set to 0.25 au, CASPT2-0.25, was used as well as the CASPT2-0 without IPEA shift. As can be seen in Table S2 of the Supporting Information, the size of the basis set does not markedly affect the relative energies of the lowest singlets and triplet states. ANO Basis sets 2 and 3 lead virtually to the same values, which differ by less than 0.1 eV when applying a def2-TZVP basis set. For this reason, in this article, only the results obtained with Basis 2 are reported. Graphical representations of the active orbitals of the monomer are included in Figure S1 of the Supporting Information together with a short description of the computation of the monomer wave functions.

To avoid the so-called IPEA dilemma,^53^ N-Electron Valence State Second-Order Perturbation Theory (NEVPT2)^54^ calculations, as implemented in OpenMolcas, have been performed. In contrast to CASPT2, NEVPT2 applies a Dyall Hamiltonian as a zeroth-order Hamiltonian, which is intruder state free and does not need parameters like the IPEA shift. The two formulations of NEVPT2, strongly contracted (SC-NEVPT2) and partially contracted (PC-NEVPT2), have been applied. However, in the present implementation in OpenMolcas, NEVPT2 calculations can be performed only with a Density Matrix Renormalization Group (DMRG) wave function as a reference, instead of a standard CASSCF wave function. Hence, DMRG calculations were carried out for all states starting from the converged CASSCF(8,8) monomer wave functions. A value of 2000 for the number of renormalized states, *m*, and a maximum number of 5 sweeps were sufficient to reproduce the CASSCF(8,8) energies.

The nonorthogonal configuration interaction for fragments (NOCI-F) method as implemented in the GronOR code^32,55^ has been used to study the dimers. PDI monomer CASSCF(8,8) wave functions (computed at the BP86-D3 optimized geometry with Basis 2) for the two lowest singlets, *S*_0_ and *S*_1_, the lowest triplet, *T*_1_, and the doublet cationic, *D*^+^, and anionic, *D*^–^, states have been employed to construct the dimer many-electron basis functions (MEBFs) as antisymmetrized spin-adapted products of the monomer wave functions, resulting in six singlet dimer MEBFs (*S*_0_*S*_0_, *S*_0_*S*_1_, *S*_1_*S*_0_, ^1^*TT*, *D*^+^*D*^–^, and *D*^–^*D*^+^). The NOCI energies and wave functions are obtained by solving the nonorthogonal 6 × 6 Hamiltonian matrix. The electronic coupling between the initial (i) and final (f) states involved in the singlet fission process is defined by the following the expression:^34^
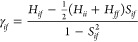
1where the initial state is a linear combination of MEBFs representing the excited singlet state and the final state is a linear combination of MEBFs corresponding to the singlet-coupled double triplet (see section 3.2.1). This method has the advantage that each electronic state of the monomer is expressed in its own optimal molecular orbitals, hence including full orbital relaxation from the start, and leads to compact wave functions, facilitating the analysis and interpretation of the results. However, this implies that the molecular orbitals of the Slater determinants that contribute to the wave function expansion are nonorthogonal to each other, making the calculations computationally much more expensive than the standard orthogonal approaches. Fortunately, the program was optimized to operate in massively parallel supercomputers, allowing NOCI calculations to be efficient and feasible for relatively large molecular systems. To reduce the dimension of the two-electron integrals, the MEBFs are expressed in a common molecular orbital basis^33^ instead of the atomic orbital basis used in the optimization of the fragment wave functions. The default threshold of 10^–5^ has been used for the removal of linearly dependent basis functions in the common molecular orbital basis of the dimer. The same threshold has been used to select the determinant pairs that are evaluated in the calculation of the nonorthogonal matrix elements. These values have been shown to be sufficient to get reliable energies and electronic couplings.^33,56^

The multiconfigurational CASSCF wave functions used to construct the MEBFs account mainly for nondynamic molecular electron correlation. The energy effects of the remaining, mainly dynamic, electron correlation are considered by shifting the diagonal elements of the NOCI matrix by a second-order perturbation theory correlation energy correction on the MEBFs.^56^ This correction is calculated from the second-order perturbation electron correlation energy for each electronic state of the monomer. Hence, from now on, we will refer to those NOCI calculations obtained by diagonalizing the Hamiltonian matrix where the MEBFs are constructed from CASSCF wave functions by NOCI(CASSCF). When the diagonal Hamiltonian matrix elements are shifted to include dynamic electron correlation, the results will be labeled with NOCI(CASPT2-0), NOCI(CASPT2-0.25), NOCI(SC-NEVPT2), or NOCI(PC-NEVPT2) depending on the specifics of the second order perturbation approach used to estimate the correlation energy correction.

## 3 Results and Discussion

### 3.1 PDI Monomer

The crystal packing of the different PDI derivatives (Figure 1) depends on the particular functional group at the N atom of the imide groups (*R*_1_). However, the absorption spectra of different PDI derivatives in solution have been shown to be almost indistinguishable from each other,^25^ indicating that the substituents do not affect the electronic properties of the PDI molecule. Hence, for simplification, the PDI molecules are usually modeled by substituting both *R*_1_ and *R*_2_ by H atoms.^28^ Here, the relative energies of the different states that are potentially involved in the SF process (*S*_1_, *T*_1_, *D*^+^ and *D*^–^) have been calculated for four different PDI derivatives. First, the simplified PDI monomer with H atoms bonded to the imide groups and, in the second place, three different substituents in the *R*_1_ position have been considered: a methyl (C1), an ethyl (C2), and an ethylphenyl group (EP).

The relative energies of the different states with respect to the *S*_0_ ground state are listed in Table 1. Results show that inclusion of the electron correlation by second-order perturbation theory has an important effect on the excitation energies, which largely stabilizes the *S*_1_ and *T*_1_ states. Compared to the experimental estimation of the excitation energies, obtained from the solution-phase absorption spectra of six PDI derivatives,^25,26^ 2.34 eV for *S*_1_ and 1.19 eV for *T*_1_, CASPT2-0.25 tends to overestimate the values while CASPT2-0 underestimates them. SC-NEVPT2 and PC-NEVPT2 give similar values; in both cases, excitation energies lie between the CASPT2-0.25 and CASPT2-0 results. The vertical excitation energies reported in Table 1 for the *S*_1_ and *T*_1_ states turn out to be smaller than those computed by DFT calculations applying the ωB97X-D functional, with values for the vertical *S*_0_ to *S*_1_ excitation of 2.84^57^ and 2.90 eV,^28^ depending on the basis sets, and 1.55 eV for the *T*_1_ excitation.^28^ Restricted active space configuration interaction with double spin-flip calculations lead to even higher excitations, with *S*_1_ ∼ 0.5 eV and *T*_1_ ∼ 0.3 eV blue-shifted compared to the ωB97X-D results.^28^ Here, except for the CASPT2-0.25 relative energies, the other second-order electron correlation corrections lead to lower excitation energies than experiment, with the PC-NEVPT2 showing better agreement with spectroscopic data,^25,26^ with values underestimated by about 0.4 eV for the *S*_1_ excitation and less than 0.2 eV for the *T*_1_ state. The *E*(*S*_1_) - 2 *E*(*T*_1_) energy difference is negative, i.e., singlet fission is not thermodynamically favorable, by −0.6 eV at the CASPT2-0.25 level of calculation and by −0.2 eV for SC-NEVPT2 and PC-NEVPT2, while the process is exoergic by 0.2 eV at the CASPT2-0 level. As can be seen in Table 1, the relative energies obtained with the three substituents, methyl, ethyl, and ethylphenyl, do not significantly differ from those obtained with the model monomer with H, meaning that the states involved in the SF process are not affected by these substituents at the *R*_1_ position. The most remarkable difference is found for the ionized doublet *D*^+^ state, which is stabilized by ∼0.2 eV when the H at the *R*_1_ position is replaced by substituents with larger electron donating character, such as the alkyl groups.

**Table 1:** Relative Energies (in eV) with Respect to the *S*_0_ State for the PDI Monomers with Different *R*_1_-Substituents Calculated by CASSCF(8,8), CASPT2-0.25, CASPT2-0, SC-NEVPT2, and PC-NEVPT2 Applying Basis 2 (See Text)

	CASSCF(8,8)	CASPT2-0.25	CASPT2-0	SC-NEVPT2	PC-NEVPT2
*R*_1_ = H					
*S*_1_	4.30	2.58	1.79	1.92	1.79
*T*_1_	2.29	1.58	0.80	1.04	0.97
*D*^+^	7.84	7.60	7.22	7.08	7.01
*D*^–^	–1.02	–2.30	–2.62	–2.80	–2.86
*R*_1_ = methyl					
*S*_1_	4.28	2.56	1.77	1.90	1.77
*T*_1_	2.27	1.58	0.79	1.04	0.97
*D*^+^	7.67	7.45	7.06	6.93	6.86
*D*^–^	–0.91	–2.19	–2.52	–2.69	–2.74
*R*_1_ = ethyl					
*S*_1_	4.28	2.55	1.76	1.89	1.76
*T*_1_	2.27	1.57	0.79	1.04	0.97
*D*^+^	7.64	7.41	7.02	6.89	6.82
*D*^–^	–0.90	–2.18	–2.51	–2.69	–2.74
*R*_1_ = ethylphenyl					
*S*_1_	4.28	2.54	1.74	1.88	1.75
*T*_1_	2.27	1.57	0.78	1.03	0.96
*D*^+^	7.66	7.42	7.0 3	6.91	6.83
*D*^–^	–0.98	–2.27	–2.59	–2.77	–2.82

### 3.2 PDI Dimer

**Figure 3:**
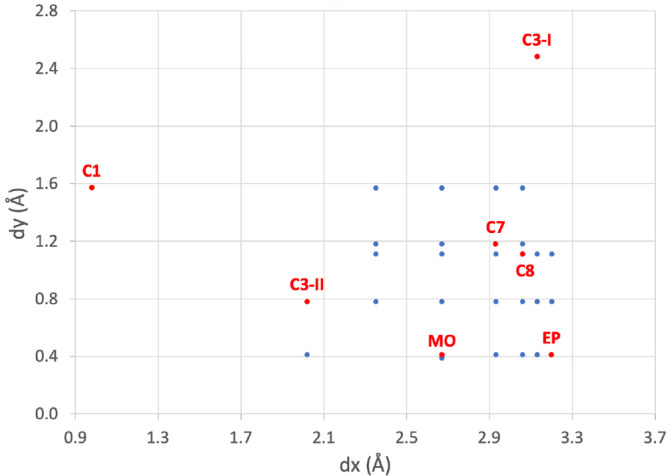
Slip-stacking displacements d*x* and d*y* for the PDI dimers considered. Labeled points in red represent geometries that have been taken from reported crystal structures.^58−60^

To stud*y* the singlet fission process in different PDI derivatives, a minimal model composed of two neighboring monomers has been applied, and various dimer conformations have been considered. First, we have studied the conformations corresponding to seven PDI derivatives for which crystallographic data is available.^58−60^ These include C1 (*R*_1_ = methyl), C3–I and C3–II (*R*_1_ = propyl) showing two different crystal structures, C7 (*R*_1_ = heptyl), C8 (*R*_1_ = octyl), EP (*R*_1_ = ethylphenyl), and MO (*R*_1_ = methoxyphenyl), while *R*_2_ = H in all cases (see Figure 1). For these materials, the model dimers were first constructed by placing the two monomers with *R*_1_ = H arranged as in the respective crystal structures. The interplane distances (dz) between monomers are 3.40 Å for C1 and C3–I, 3.41 Å for C3–II, 3.50 Å for C7, 3.28 Å for C8, 3.48 Å for EP, and 3.46 Å for MO. Furthermore, to analyze the dependence of the electronic properties of the PDI dimers with the d*x* and d*y* slip-stack displacements (Figure 2), 23 configurations have been generated in the region where most of the experimental PDI derivatives are found. All those configurations are placed at d*z* = 3.48 Å, the one corresponding to the EP derivative, which is one of the candidates that has been suggested to exhibit SF. Notice that for most of the PDI derivatives, the separation in the perpendicular *z*-direction is very similar, around 3.40–3.50 Å.^58−60^Figure 3 shows the whole set of conformations scanned, disclosing the geometries extracted from the crystal structures.

For the entire set of dimer geometrical conformations, NOCI calculations have been performed in order to construct the NOCI wave functions of the six lowest electronic states, analyze their character, and determine both the relative energies between the different dimer states and the electronic couplings. As commented above, one of the main advantages of the NOCI approach is the simple interpretation of the wave functions, since they are written in a short expansion of MEBFs with recognizable character. For all of the dimers, the NOCI wave functions have been analyzed. As an example, Table 2 shows the expansion of the NOCI wave functions in terms of the MEBFs for the PDI dimer in the EP orientation, computed at the CASSCF level and by diagonalizing the NOCI matrix including the electron correlation correction by CASPT2-0.25, CASPT2-0, and PC-NEVPT2 (SC-NEVPT2 results are not reported since they are virtually identical with those obtained by PC-NEVPT2). The relative energies of the NOCI states are also collected in Table 2. Similar NOCI wave functions are obtained for the different calculations performed (with and without dynamic electron correlation correction), although the energetic ordering of the states can be slightly different. As shown in Table 2, the NOCI wave functions show important mixing between the different MEBFs, except for the lowest electronic state, Ψ_1_, which corresponds to the ground state *S*_0_*S*_0_ configuration. For instance, for the NOCI(CASSCF), Ψ_3_ is a combination of *S*_0_*S*_1_ and *S*_1_*S*_0_ MEBFs with similar weights, so-called excitonic resonance (ER) states, while Ψ_2_ is a combination of ER states, with charge transfer resonance (CR) states, mixtures of *D*^+^*D*^–^ and *D*^–^*D*^+^ charge transfer states between the two monomers. Ψ_4_ has a large contribution from the ^1^*TT* state, with important weights of CR states. Ψ_5_ and Ψ_6_ have large CR contributions, with sizable mixing with ^1^*TT* or ER states, respectively. These large mixtures between different states were already pointed out by Farag and Krylov^28^ in their study of SF in PDI dimers, based on restricted active space configuration interaction method with double spin-flip (RAS-2SF). This is in contrast to that found in other SF systems like acenes, diphenylisobenzofuran derivatives or diazadiborinine,^10,11,56,61^ where the wave functions appear to be more localized in few dimer states. On the other hand, the CASPT2-0 corrected NOCI wave functions slightly differ from the NOCI(CASSCF), NOCI(CASPT2-0.25) and NOCI(NEVPT2) wave functions. In particular, the lowest excited state, Ψ_2_, is mainly of ^1^*TT* character, with small contributions from CR states. The lack of IPEA correction in NOCI(CASPT2-0) overstabilizes the ^1^*TT* state, which now lies below the *S*_0_*S*_1_ ± *S*_1_*S*_0_ states. The energy gap of the ^1^*TT* and *D*_0_D_1_ ± *D*_1_D_0_ states also increases, which significantly reduces the mixing among them in the final NOCI wave functions.

**Table 2:** MEBF Coefficients and Relative Energies (eV) of the Six NOCI Wavefunctions for a PDI Dimer at the EP Conformation with *R*_1_ = H^a^

MEBF	Ψ_1_	Ψ_2_	Ψ_3_	Ψ_4_	Ψ_5_	Ψ_6_
NOCI(CASSCF)						
Δ*E*	0.00	4.26	4.36	4.52	5.09	5.10
*S*_0_*S*_0_	**0.996**	–0.002	0.040	0.061	0.064	0.002
*S*_0_*S*_1_	–0.020	**0.562**	**0.695**	–0.116	–0.025	**-0.433**
*S*_1_*S*_0_	0.020	**0.557**	**-0.698**	0.116	0.028	**-0.434**
^1^*TT*	–0.004	0.000	0.094	**0.719**	**-0.690**	–0.002
*D*^+^*D*^–^	0.039	**0.405**	–0.094	**-0.460**	**-0.530**	**0.579**
*D*^–^*D*^+^	–0.039	**0.406**	0.092	**0.460**	**0.527**	**0.582**
NOCI(CASPT2-0.25)						
Δ*E*	0.00	2.61	2.66	3.07	3.53	3.64
*S*_0_*S*_0_	**0.991**	–0.003	0.053	–0.097	0.002	–0.079
*S*_0_*S*_1_	–0.032	**0.608**	**0.699**	0.043	**-0.373**	0.017
*S*_1_*S*_0_	0.034	**0.594**	**-0.711**	–0.043	**-0.374**	–0.016
^1^*TT*	–0.008	0.000	0.023	**-0.669**	0.001	**0.774**
*D*^+^*D*^–^	0.063	**0.342**	–0.040	**0.506**	**0.620**	**0.490**
*D*^–^*D*^+^	–0.063	**0.344**	0.033	**-0.505**	**0.619**	**-0.491**
NOCI(CASPT2-0)						
Δ*E*	0.00	1.73	1.86	1.89	2.73	2.82
*S*_0_*S*_0_	**0.986**	0.072	–0.004	–0.066	–0.142	0.003
*S*_0_*S*_1_	–0.045	0.033	**0.627**	**-0.693**	0.024	**-0.352**
*S*_1_*S*_0_	0.049	–0.031	**0.601**	**0.716**	–0.025	**-0.352**
^1^*TT*	–0.021	**0.948**	–0.003	0.055	**0.313**	–0.001
*D*^+^*D*^–^	0.085	**-0.195**	**0.321**	0.012	**0.677**	**0.629**
*D*^–^*D*^+^	–0.084	**0.196**	**0.321**	0.000	**-0.673**	**0.633**
NOCI(PC-NEVPT2)						
Δ*E*	0.00	1.73	1.89	1.92	2.48	2.53
*S*_0_*S*_0_	**0.981**	–0.003	0.137	–0.076	0.120	0.004
*S*_0_*S*_1_	–0.045	**0.516**	**0.574**	**0.408**	–0.022	**-0.486**
*S*_1_*S*_0_	0.049	**0.512**	**-0.577**	**-0.408**	0.028	**-0.487**
^1^*TT*	–0.020	0.002	**0.401**	**-0.609**	**-0.685**	–0.004
*D*^+^*D*^–^	0.104	**0.459**	**-0.275**	**0.370**	**-0.532**	**0.534**
*D*^–^*D*^+^	–0.103	**0.462**	**0.271**	**-0.370**	**0.524**	**0.542**

a In bold, coefficients larger than 0.15.

In some cases, two states with sizable and almost equal contributions from the ^1^*TT* MEBF are found. For instance, in the NOCI(CASSCF) wave functions for the EP dimer (see Table 2), Ψ_4_ and Ψ_5_ show similar coefficients in the ^1^*TT* MEBF. Hence, in this case, the state that is lowest in energy has been considered to represent the situation where the two monomer triplet states are coupled to singlet. However, sometimes a higher electronic state shows a larger coefficient in the ^1^*TT* MEBF than a lower-lying state, as found in the NOCI(CASPT2-0.25) wave functions, where Ψ_6_ shows a somewhat larger coefficient in the ^1^*TT* MEBF than Ψ_4_. In these cases, as the difference in weights is not substantial, the lowest Ψ_4_ state has been chosen as the reference for the ^1^*TT* state. Farag and Krylov^28^ found a similar behavior for some PDI dimers (EP, MO, C3–I, C7 and C8). However, in their calculations, the higher state had a much larger ^1^*TT* character that the lower one and the two possible ^1^*TT* states were explored in their work.

Among the different MEBFs considered in the NOCI, the CT configurations imply the movement of electrons among the monomers. Therefore, these configurations are, in principle, most susceptible to polarization effects induced by the environment. Bellinger et al. have reported a detailed study of the relative energies of the Frenkel excitons (*S*_0_*S*_1_ ± *S*_1_*S*_0_) and the CT states as a function of different representations of the environment of a PDI dimer.^62^ As the most relevant conclusion in the scope of the present study, they revealed that the relative energy of the symmetric CT states, i.e., those that are mainly described by the *D*^+^*D*^–^ ± *D*^+^*D*^–^ configurations, is hardly affected by inclusion of solvent effects. They found a substantial lowering of the energy only when the CT state distorts to its optimal geometry, associated with a collapse of the wave function in either the *D*^+^*D*^–^ or *D*^–^*D*^+^ configuration. As shown in Table 2, the contribution of the CT configurations to the electronic states is symmetric in all cases, which means that the polarization effects in the environment are expected to be small. Moreover, it should be realized that the CT states are never populated in the SF process in PDI, they only contribute to the multiconfigurational wave function of the states involved in the SF process. Therefore, here, geometry distortions are not at play.

The singlet fission process starts by exciting one PDI molecule from the ground state to the lowest singlet excited state. In a dimer, this situation is described as an exciton resonance state of *S*_0_*S*_1_ ± *S*_1_*S*_0_ character. Calculations of the NOCI oscillator strengths from the initial *S*_0_*S*_0_ ground state for the various PDI dimer conformations, show that the state that carries intensity by optical absorption corresponds to *S*_0_*S*_1_ + *S*_1_*S*_0_ with an oscillator strength of ∼1.6 au The *S*_0_*S*_1_ – *S*_1_*S*_0_ combination is the dark state, showing an oscillator strength of the order of 1 × 10^–5^ au. The bright state turns out to be the lowest electronic state for dimer conformations with small displacements along the short axes, d*y* ≤0.41 Å, (EP and MO among them), while for the rest of the conformations explored, the *S*_0_*S*_1_ – *S*_1_*S*_0_ is lower in energy (see as an example Table S3 of the Supporting Information where the NOCI wave functions for the C7 dimer conformation are detailed). The energetic difference between these two states for all conformations is usually small, on the order of 0.1 eV. Although the *S*_0_*S*_1_ + *S*_1_*S*_0_ state is the bright state, the electronic coupling of this state with the state of mainly ^1^*TT* character is small. Instead, the coupling between the *S*_0_*S*_1_ – *S*_1_*S*_0_ and ^1^*TT* states is much larger (as will be shown below), thus favoring the SF process. As the two states, *S*_0_*S*_1_ + *S*_1_*S*_0_ and *S*_0_*S*_1_ – *S*_1_*S*_0_, are singlets and lie close in energy, it is expected that both are accessible after irradiation. Hence, the *S*_0_*S*_1_ – *S*_1_*S*_0_ state, which shows larger coupling with the ^1^*TT* state, is taken as a reference to compute the SF energies and electronic couplings.

In Table 3 the SF energy, defined as *E*_*SF*_ = *E*(^1^*TT*) – *E*(*S*_0_*S*_1_ – *S*_1_*S*_0_) is reported. A positive value stands for an endoergic process, while a negative value means an exoergic process, with the ^1^*TT* state lower in energy than the initial *S*_0_*S*_1_ – *S*_1_*S*_0_ state. Results in Table 3 reveal that NOCI(CASSCF) calculations show an endoergic process for all of the systems. Inclusion of correlation energy correction in the NOCI calculations by the standard CASPT2-0.25 method increases these values while NOCI(CASPT2-0) leads to exoergic energies of around 0.1 eV, which means that singlet fission would be favorable. SC-NEVPT2 and PC-NEVPT2 energy corrections in the NOCI calculations have also been considered, both resulting in very similar values. Singlet fission NOCI(NEVPT2) energies are in between the values obtained by NOCI(CASPT2) with and without IPEA shift. These results are in line with those obtained for the monomer, showing that NOCI(CASPT2-0) tends to overstabilize the triplet states in the monomers. Comparing the singlet fission energy of the different dimers with the corresponding energy difference on the monomer, 2*E*(*T*_1_) – *E*(*S*_1_), it turns out that the process is energetically more favorable in the EP and MO dimers than in the monomer, while it is less favorable for the rest of conformations listed in Table 3. From these results, the conformations corresponding to the EP and MO systems fulfill the thermodynamic requirement, with *E*_*SF*_ negative or slightly positive, depending on the particular electron correlation correction included, and hence, from the energetic point of view, they are the most likely candidates to undergo SF.

**Table 3:** Singlet Fission Energies (in eV), *E*_*SF*_, for the PDI Dimers (with *R*_1_ = H) for Which the Crystal Structure Is Available

	NOCI(x)				
	x = CASSCF	x = CASPT2-0.25	x = CASPT2-0	x = SC-NEVPT2	x = PC-NEVPT2
EP	0.16	0.41	–0.16	0.04	0.03
MO	0.26	0.48	–0.14	0.18	0.17
C1	0.38	0.60	0.04	0.26	0.26
C3–I	0.40	0.65	–0.06	0.27	0.26
C3–II	0.49	0.78	–0.02	0.38	0.37
C7	0.45	0.68	–0.06	0.35	0.35
C8	0.43	0.63	–0.11	0.36	0.36
monomer	0.19	0.59	–0.19	0.16	0.15

**Figure 4:**
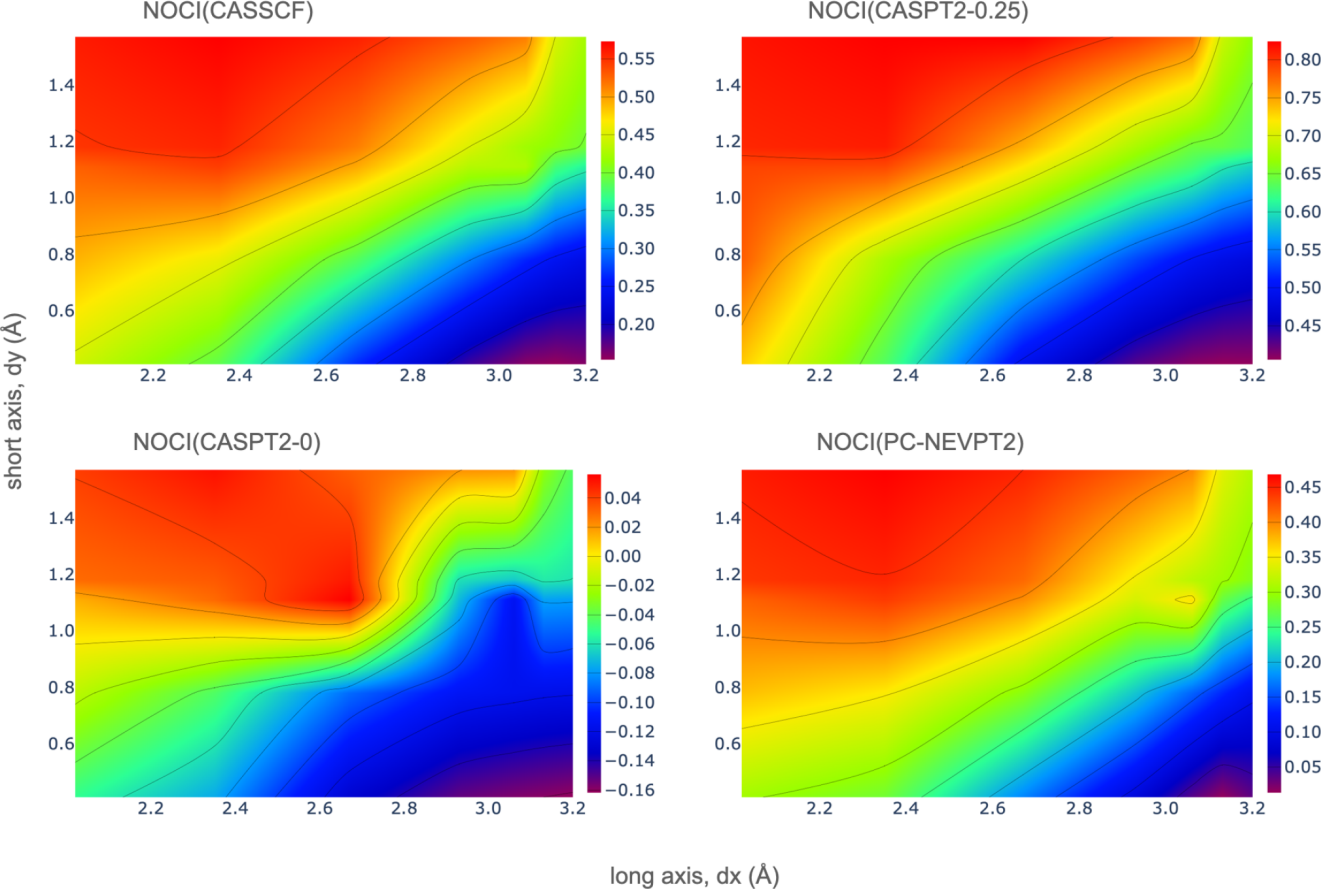
Singlet fission energy (eV), *E*_*SF*_, computed by NOCI calculations, as a function of the d*x* and d*y* displacements for PDI dimers with *R*_1_ = H.

To investigate the dependency of the singlet fission energy on the d*x* and d*y* slip-stack displacements, apart from the seven PDI experimental structures, as commented on before, 23 additional dimer conformations have been studied. Figure 4 shows the variation of the singlet fission energy with the displacements along the long *x* and the short *y* axes as heat maps to facilitate the interpretation. Small positive or negative values favor the singlet fission process. It can be seen that all levels of the NOCI calculations, CASSCF, CASPT2-0.25, CASPT2-0 and PC-NEVPT2, coincide in pointing to large *x* displacements (d*x* ≥ 2.6 Å) and short *y* values (d*y* ≤ 0.8 Å) as the energetically most favorable region for SF (blue-violet region in Figure 4). From the seven PDI derivatives reported experimentally, the EP and MO dimers display slip distances in d*x* and d*y* that meet this criterion.

#### 3.2.1 Electronic Couplings

The electronic coupling between the *S*_0_*S*_1_ – *S*_1_*S*_0_ state and the state where the two monomer triplets are coupled to a singlet, ^1^*TT*, has been computed by equation 1 in two different ways. First, it is a direct electronic coupling between the *S*_0_*S*_1_ – *S*_1_*S*_0_ and the ^1^*TT* MEBFs. In the second place, the coupling is computed allowing for a charge transfer enhanced mechanism, in which the effect of the charge transfer excitations between the two monomers is included. This is done by first diagonalizing the *S*_0_*S*_1_, *S*_1_*S*_0_, *D*^+^*D*^–^, and *D*^–^*D*^+^ 4 × 4 Hamiltonian matrix leading to new MEBFs. Two eigenvectors are dominated by the *S*_0_*S*_1_ ± *S*_1_*S*_0_ combination of the original MEBFs, now dressed with *D*^+^*D*^–^ ± *D*^–^*D*^+^ contributions. Similarly, a dressed ^1^*TT* state is obtained from a 3 × 3 Hamiltonian diagonalization including the ^1^*TT*, *D*^+^*D*^–^ and *D*^–^*D*^+^ MEBFs. After reexpressing the NOCI Hamiltonian and overlap matrix in this new set of MEBFs, the total electronic couplings between the *S*_0_*S*_1_ – *S*_1_*S*_0_ and ^1^*TT* states can be calculated using equation 1. The total coupling accounts for both, the direct coupling and the charge transfer enhanced contribution to the coupling. Charge transfer effects on the coupling are expected to be important since the final NOCI wave functions showed large mixing of states of different nature; particularly, ^1^*TT* and *S*_0_*S*_1_ ± *S*_1_*S*_0_ substantially mix with CR states (see Table 2). The importance of the involvement of charge transfer states in the singlet fission process has been previously pointed out in tetracene^63^ and PDI derivatives.^30,64^

In Table 4 the calculated electronic couplings are reported. The total coupling is computed by NOCI(CASSCF) calculations and including the electron correlation energy correction as commented before. In the latter case, the charge transfer dressed MEBFs are constructed by diagonalizing the shifted Hamiltonian. Instead, results of the direct coupling are only shown for the NOCI(CASSCF) calculations since inclusion of the electron correlation energy correction, either by CASPT2-0.25, CASPT2-0 or NEVPT2, does not significantly affect the couplings when charge transfer states are not incorporated.

**Table 4:** Direct and Total Electronic Couplings (in meV) between ^1^*TT* and *S*_0_*S*_1_ – *S*_1_*S*_0_ for the PDI Dimers (with *R*_1_ = H) for Which the Crystal Structure Is Available

	NOCI(x) direct	NOCI(x) total				
	x = CASSCF	x = CASSCF	x = CASPT2-0.25	x = CASPT2-0	x = SC-NEVPT2	x = PC-NEVPT2
EP	3.17	29.27	26.34	18.53	36.40	36.32
MO	5.46	55.54	52.87	30.15	65.79	65.50
C1	3.74	56.35	56.86	22.48	62.65	62.26
C3–I	1.64	43.33	52.43	14.58	49.90	48.69
C3–II	2.31	24.99	6.83	6.16	11.50	14.04
C7	5.85	81.45	92.71	27.43	90.95	89.09
C8	10.95	110.33	111.21	53.38	122.01	120.98

As can be extracted from Table 4, direct couplings are small in all systems. A somewhat larger value is found for the C8 dimer, for which the interplanar distance is a bit shorter than that in the other dimers. Inclusion of charge transfer configurations to describe the ^1^*TT* and *S*_0_*S*_1_ – *S*_1_*S*_0_ states largely increases the magnitude of the coupling, by at least 1 order of magnitude. NOCI(CASSCF) values are similar to those obtained by NOCI(CASPT2-0.25) and NOCI(NEVPT2), while NOCI(CASPT2-0) total electronic couplings are significantly smaller as a consequence of the lesser mixing of the ^1^*TT* with the charge transfer MEBFs. Note that the systems for which the thermod*y*namic criterion was more favorable to experience SF (EP and MO) do not show the largest coupling. The largest couplings are found for dimer conformations corresponding to the C7 and C8 PDI compounds, while the smaller is observed for the C3–II PDI conformation.

**Figure 5:**
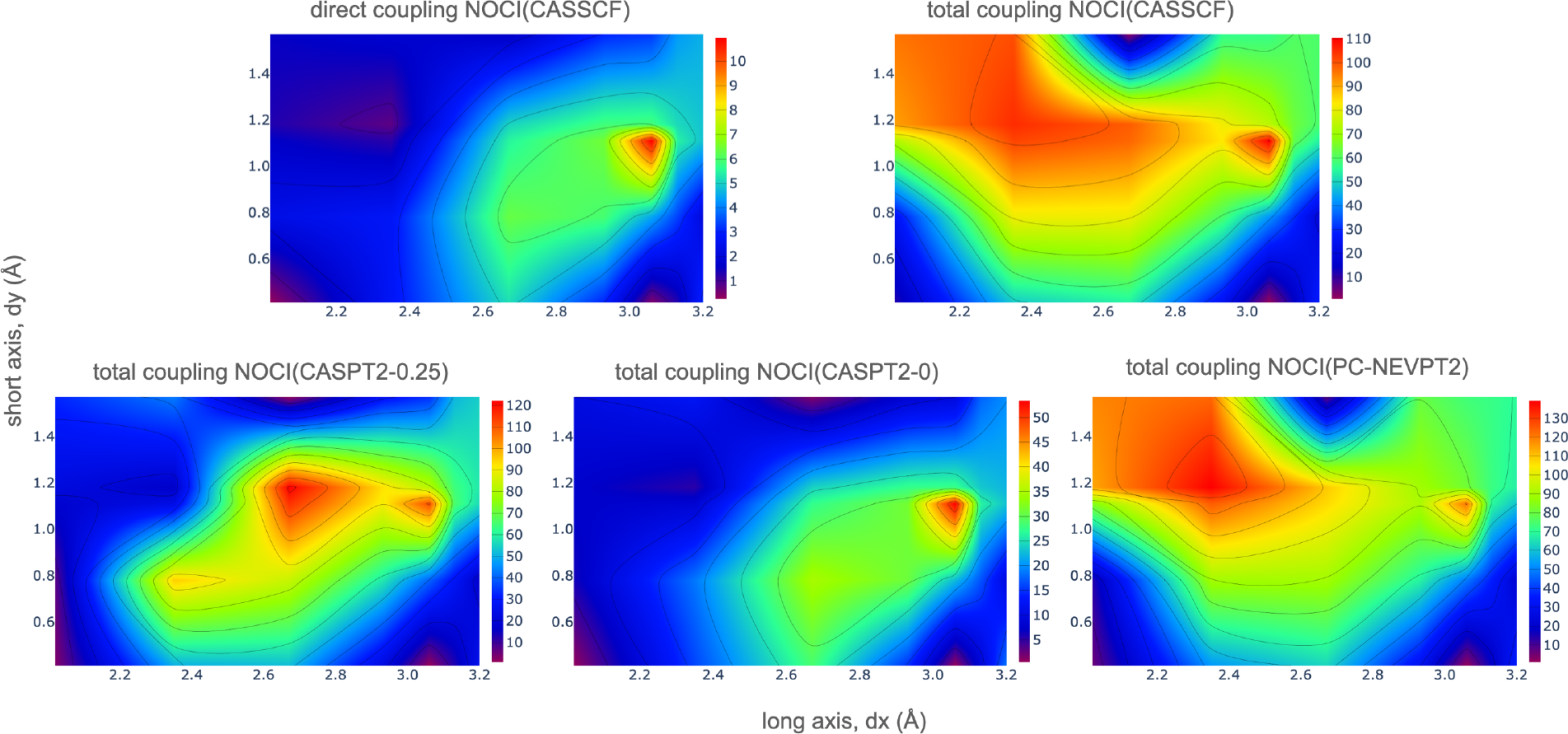
Electronic coupling (meV) between the ^1^*TT* and *S*_0_*S*_1_ – *S*_1_*S*_0_ states, computed by NOCI calculations, as a function of the d*x* and d*y* displacements between two PDI molecules with *R*_1_ = H.

In Figure 5, the electronic couplings, both direct and total, between the ^1^*TT* and *S*_0_*S*_1_ – *S*_1_*S*_0_ states are plotted as a function of the d*x* and d*y* displacements. The region with larger electronic couplings differs from that found for the favorable *E*_*SF*_ energy. Although the magnitude of the couplings is very different, the heat map for the NOCI(CASSCF) direct coupling closely resembles the one obtained for the total coupling NOCI(CASPT2-0) calculations, with a small region of maximum values at d*x* ≥ 3.0 Å and d*y* ∼ 1.0–1.2 Å. This can be explained by the earlier discussed tendency of NOCI(CASPT2-0) to overstabilize the ^1^*TT* state, leading to very little interaction with charge transfer states. This region of high couplings is also present when the charge transfer effects are incorporated in the total electronic couplings, computed by NOCI(CASSCF), NOCI(CASPT2-0.25) and NOCI(PC-NEVPT2). However, in these calculations, a new region of large couplings is found at intermediate d*x* distances and d*y* ∼1.0–1.2 Å. NOCI(CASSCF) and NOCI(PC-NEVPT2) total coupling heat maps turn out to be very similar, with a region of large couplings that expands from d*x* ∼ 2.2–2.6 Å. The difference between these two heat maps and the one obtained by a CASPT2-0.25 energy correction is due to the fact that different dimer conformations studied at d*x* = 2.35 Å do present two states with significant ^1^*TT* character. In the CASSCF and NEVPT2 NOCI calculations the lowest state shows a sizable ^1^*TT* contribution and has been taken as reference although a higher state with slightly larger ^1^*TT* coefficient exists. Instead, in the NOCI wave functions obtained including the CASPT2-0.25 correction, the coefficient of the lowest state with ^1^*TT* character is too small (≤0.3) to be considered a reasonable description of the ^1^*TT* state, and therefore, a higher state with larger ^1^*TT* character has been considered to evaluate the electronic couplings. Hence, the higher relative energy of ^1^*TT* leads to small couplings in this region. Overall, the results show that charge transfer states play an important role in the mechanism of SF in PDI dimers since the mixing of the ^1^*TT* with CR states significantly strengthens the electronic coupling. Moreover, heat maps reveal that, regardless of the method, the couplings are more favorable at relatively large d*x* and intermediate d*y* displacements.

Up to this point, the outcome of the two main properties to describe the SF process slightly differ. The thermodynamic criterion for singlet fission is accomplished for large displacements in the *x* direction and short *y* shifts, while the electronic coupling is bigger for rather large d*x* and intermediate d*y* displacements. However, none of these two properties by itself can be taken as unique reliable descriptor for optimal SF;^25,28^ instead, a combination of both relative energies and electronic coupling would be more suitable to establish the more favorable conformations for SF to occur. In this sense, kinetic models allow combining both descriptors and become a better tool to assess the feasibility of the process.

#### 3.2.2 Relative Singlet Fission Rates

**Figure 6:**
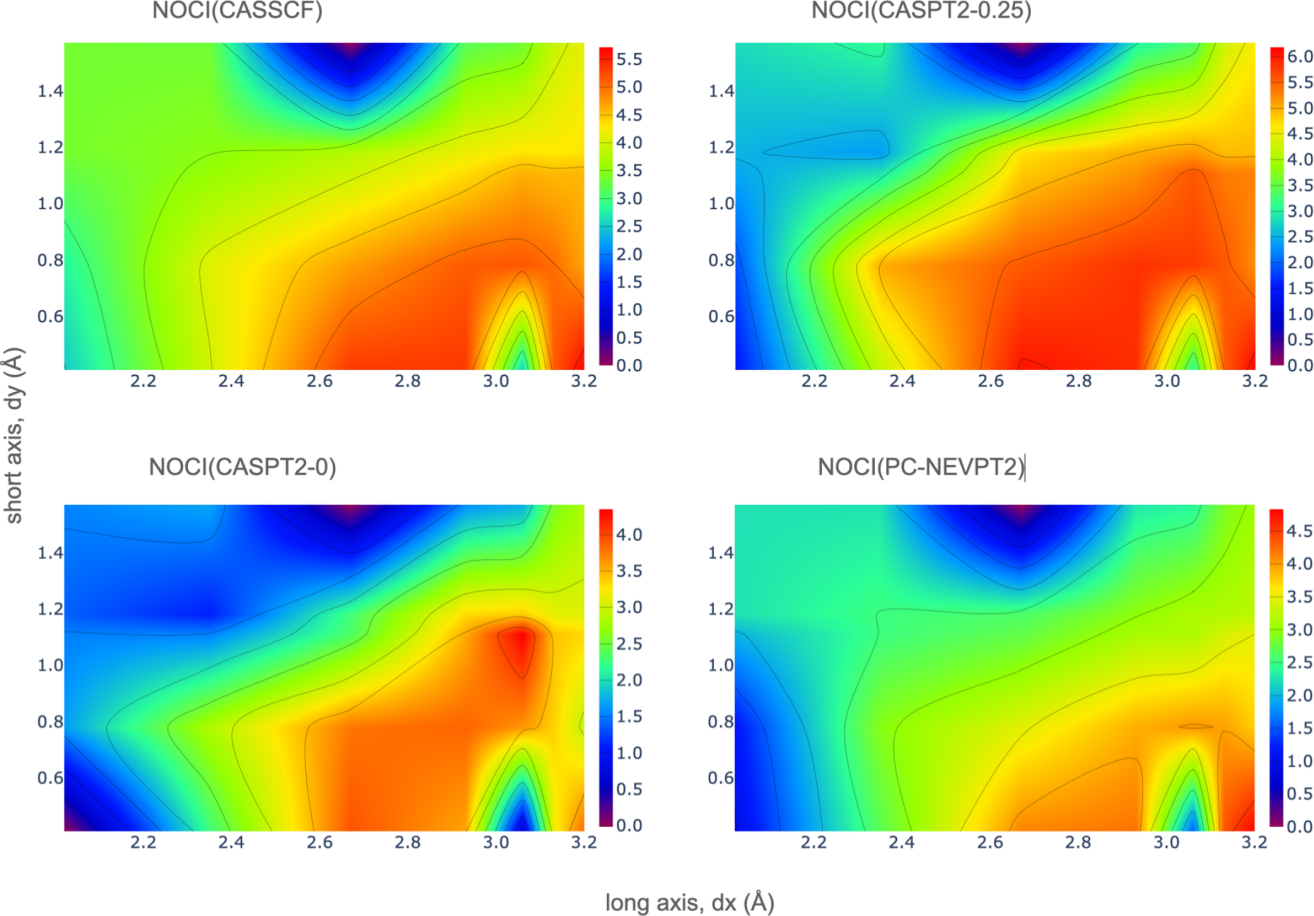
log of the relative singlet fission rates, computed by NOCI calculations, as a function of the d*x* and d*y* displacements for PDI dimers with *R*_1_ = H.

Here, we follow the kinetic model proposed by Krylov and co-workers,^10,28,65^ according to which singlet fission occurs in two steps: the first step corresponds to the transition from the initial photoexcited state, here *S*_0_*S*_1_ – *S*_1_*S*_0_, to the ^1^*TT*, while the second step is the decoupling and separation of the two triplets. The rate of the first step of the SF process, *r*_1_, can be estimated by the expression used in reference (28):
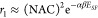
2where NAC reads for nonadiabatic coupling between the *S*_0_*S*_1_ – *S*_1_*S*_0_ and ^1^*TT* states. Here, it is computed as the total intermolecular electronic coupling. The parameter α is set to 0.5 as suggested by Kolomeisky et al.,^66^ assuming that the transition state is placed halfway along the reaction coordinate between reactants and products, β =  with *k* the Boltzmann constant, *T* the temperature, typically *T* = 300 K, and *E*_*SF*_ the SF energy as defined before. Notice that the rate, *r*_1_, leads to faster processes for large electronic couplings and exothermic or small endothermic SF energies and therefore combines the two main descriptors of the SF process. This approach, however, only allows to compute relative rates in a series of similar compounds, but cannot provide absolute rate values. Here, we have computed the rates with respect to the lowest value and represent the decimal logarithm of the relative singlet fission rates as a function of the *x* and *y* variation; see Figure 6. The relative rates have been calculated from the total electronic couplings and singlet fission energies, computed by NOCI(CASSCF), NOCI(CASPT2-0.25), NOCI(CASPT2-0), and NOCI(PC-NEVPT2) calculations, as previously described. From Figure 6, it can be seen that all of the methods result in similar heat maps. Larger values mean kinetically more favored SF process. These can be found in a region with d*x* ∼2.6–2.9 Å and d*y* below ∼0.9 Å. It is also remarkable that the rate becomes significant again at d*x* ∼ 3.2 Å and d*y* ≤ 0.5 Å, which is compatible with the conformation of the EP dimer. The region around d*x* ∼ 3.05 Å and d*y* ≤ 0.5 Å shows a slow rate, which can be explained by the low electronic couplings in this region, as shown in Figure 5. Regarding the seven derivatives experimentally characterized, the relative rates for the formation of the ^1^*TT* state obtained here posit the EP conformation as the more favorable for singlet fission, followed by the MO conformation, C1, C31, and C8 with similar values, and C32 as the most unfavorable arrangement. These results are in accordance with the relative rates reported by Farag et al.,^28^ showing the MO derivative as the kinetically most favorable for SF, closely followed by the EP, while the relative rates of the C1, C8 and C31 derivatives remain very similar. These results also concur with the outcomes from time-resolved emission experiments^25^ that point to the MO and EP PDI derivatives as the systems undergoing fastest SF, the former within 231 ps and 173% yield, and the second around 260 ps and 178% SF yield, while the rate is 1 order of magnitude slower for the C8 PDI derivative.

All in all, the results suggest that small displacements along the short axis *y* and large displacements along the long axis *x* are more favorable for the formation of the ^1^*TT* state and, thus, to singlet fission. These findings are in agreement with previous works by Mirjani et al.,^30^ who suggested a d*y* = 0 and d*x* ∼ 2.8 Å as the optimal geometry, and by Le et al.,^25^ who indicated a slip along the long axis d*x* ∼ 3.0 Å and minimal short axis displacement. In contrast, Farag et al.^28^ postulate displacements ≥2.5 Å for both *x* and *y* axes.

The rate of the second step of the SF process, the separation of the dimer ^1^*TT* state into two decoupled triplets, is related to the ^1^*TT* binding energy, *E*_*b*_, that can be estimated by the quintet state approximation as the energy difference between the dimer quintet state, ^5^*TT*, and the ^1^*TT* state, *E*_*b*_ = *E*(^5^*TT*) – *E*(^1^*TT*).^7^ Notice that the ^5^*TT* state has a pure diabatic *TT* character and therefore, gives a reasonable estimate of the energy of two isolated triplets, while the dimer ^1^*TT* state mixes with charge resonance states, as discussed before. As shown in Table 5, the separation of the ^1^*TT* state into two triplets is not energetically favored, and the energy needed varies depending on the conformation, from ∼60 meV for the C3–II conformation to ∼320 meV for the EP structure. As previously found for the relative energies, here the binding energies obtained by NOCI(NEVPT2) lie in between the values obtained by NOCI(CASPT2-0.25) and NOCI(CASPT2-0), the later leading to the lowest values. The mixing with charge resonance states stabilizes the ^1^*TT* state, favoring exciton trapping with respect to triplet separation. Inclusion of the CASPT2-0 correction tends to localize the ^1^*TT* state, which shows much less mixing with charge transfer states, and therefore, the energy needed to separate the triplets becomes smaller.

**Table 5:** ^1^*TT* Binding Energies (in eV), *E*_*b*_, for the PDI Dimers for Which the Crystal Structure Is Available

	NOCI(x)				
	x = CASSCF	x = CASPT2-0.25	x = CASPT2-0	x = SC-NEVPT2	x = PC-NEVPT2
EP	0.34	0.38	0.17	0.33	0.32
MO	0.26	0.32	0.14	0.24	0.23
C1	0.20	0.27	0.06	0.20	0.20
C3–I	0.11	0.14	0.07	0.11	0.11
C3–II	0.07	0.06	0.06	0.07	0.07
C7	0.11	0.16	0.09	0.11	0.10
C8	0.18	0.25	0.15	0.16	0.15

Comparing the various derivatives, it can be observed that conformations with a large rate for the ^1^*TT* formation, such as EP or MO, tend to display energetically unfavorable triplet separation, and consequently, the charge generating process could be hindered by the second step. In contrast, C3–II shows the lowest rate since the interaction of the ^1^*TT* MEBF with charge resonance states is limited, and as a result, triplet separation is energetically more likely. Clearly, to describe the mechanism of the triplet separation process, the estimation of the ^1^*TT* binding energy is not sufficient, and the different competing relaxation pathways have to be considered, which is out of the scope of the present work. Still, the fact that the triplet separation is not energetically favored is in accordance with the fast decay of the ^1^*TT* to the ground state observed experimentally.^25^

#### 3.2.3 Effect of the *R*_1_ Substituent in the Dimers

As shown before, the specific substituent at the imide group, *R*_1_, has no influence on the relative energies of the electronic states of the PDI monomer. Based on this fact, most computational studies model the *R*_1_ substituent by a hydrogen atom using the relative orientation of the monomers as found in the crystal structure to differentiate the derivatives. Here, we quantitatively explore the effects of the *R*_1_ substituent in the properties analyzed, i.e., the singlet fission energy, the electronic couplings and the relative rates for the formation of the ^1^*TT* state. To start, we have studied the PDI dimer in the relative orientation of the EP PDI derivative considering four possible *R*_1_ groups: an H atom, a methyl, an ethyl, and the real ethylphenyl group. The relative energies of the six NOCI states for the four models of the EP dimer are very similar, with only small differences of ∼0.05 eV (see Table S4 of the Supporting Information). Singlet fission energies, *E*_*SF*_, are also similar in all cases, with values of 0.18, 0.43, −0.16, and 0.04 eV for *R*_1_ = H at the NOCI(CASSCF), NOCI(CASPT2-0.25), NOCI(CASPT2-0), and NOCI(PC-NEVPT2) level of calculation and 0.19, 0.46, −0.15, and 0.10 eV, respectively, for *R*_1_ = ethylphenyl. This behavior is general, as shown in the heat maps of the *E*_*SF*_ with respect to the *x* and *y* displacements for the dimer represented with *R*_1_ = ethyl reported in Figure S2 of the Supporting Information as an example, showing similar trends as those obtained by *R*_1_ = H (see Figure 4), thus indicating that the substituent does not significantly affect the singlet fission energy.

In contrast, the electronic couplings, as reported in Table 6, are more sensitive to the specific substituent, and significant differences appear with values that are around two times larger when *R*_1_ is substituted by the real ethylphenyl group in the EP structure in place of H. From Table 6 it can be observed that the electronic couplings for the EP dimer are very similar for H and methyl at the *R*_1_ position; however, for the ethyl substituent, which is closer to the ethylphenyl group, an important increment of the couplings is observed. The couplings obtained by the ethyl substituent are much closer to those obtained for the real ethylphenyl system, which is an indication that the ethyl group, different from the H or methyl, better represents the EP derivative. This is an interesting result since it shows that, although the substituent does not affect the singlet fission energy, it has an impact on the electronic couplings.

**Table 6:** Direct and Total Electronic Couplings (in meV) between ^1^*TT* and *S*_0_*S*_1_ – *S*_1_*S*_0_ for the PDI Dimer at the EP Conformation with *R*_1_: H, Methyl (C1), Ethyl (C2), and Ethylphenyl (EP)

	NOCI(x) direct	NOCI(x) total				
	x = CASSCF	x = CASSCF	x = CASPT2-0.25	x = CASPT2-0	x = SC-NEVPT2	x = PC-NEVPT2
*R*_1_ = H	3.17	29.27	26.34	18.53	36.40	36.32
*R*_1_ = C1	3.39	30.88	27.28	19.26	37.29	37.18
*R*_1_ = C2	5.56	55.42	49.68	32.86	64.92	64.71
*R*_1_ = EP	5.79	52.50	48.30	32.84	60.21	59.90

**Figure 7:**
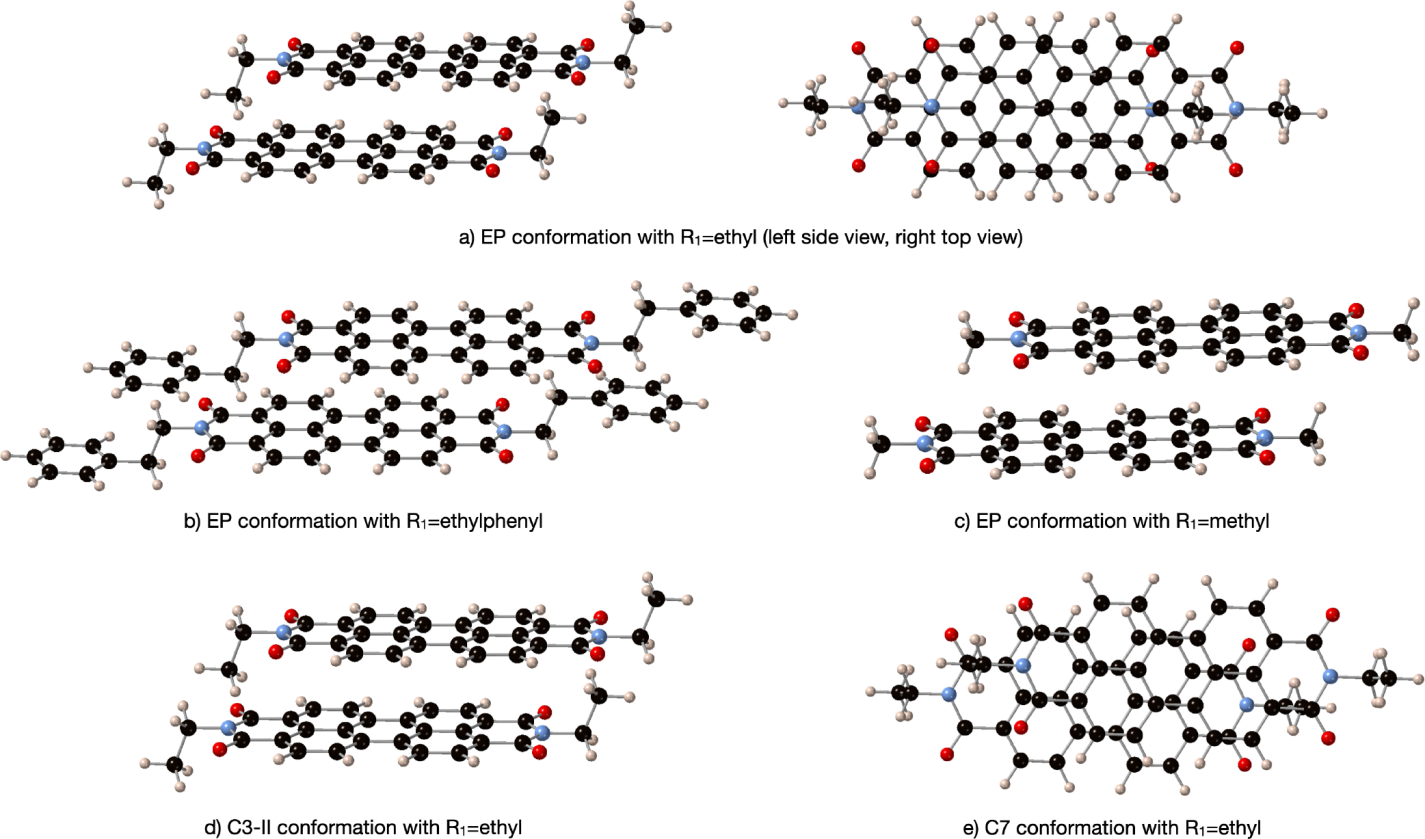
Representation of various dimer conformations.

In light of these results, we computed the electronic couplings of all of the PDI derivatives building the dimers based on a monomer with an ethyl group at the *R*_1_ position. The aim is to discern whether the ethyl substituent, which apparently better mimics alkyl groups as propyl (C3–I and C3–II dimers), heptyl (C7) or octyl (C8) than the bare H-atom, has an effect on the electronic couplings in these PDI derivatives. The optimized structure of the monomer with *R*_1_ = ethyl was used to construct the dimers. Comparing the electronic couplings obtained with *R*_1_ = H (Table 4) and *R*_1_ = ethyl (Table 7) one can see that for the C1, C3–I, C3–II and C7 dimer conformations the variation is small; the values do not change by more than 5–7 meV, and for C8 the changes are also moderate, with an 11 meV decrease being the maximum change. These small changes do not alter the relative values of the couplings. However, for the EP conformation, replacing the H-atom at the *R*_1_ position by an ethyl group remarkably increases the magnitude of the couplings, ∼30 meV, practically doubling the values obtained for *R*_1_ = H, while for the MO conformation the coupling decreases by more than ∼15 meV. This reverse trend exchanges the size of the electronic couplings, which are larger for the MO conformation compared to EP with *R*_1_ = H and smaller when *R*_1_ = ethyl. The origin of this effect must be ascribed to crystal packing effects because the same replacement does not affect the electronic structure of the monomer, as illustrated in Table 1. The EP and MO conformations show the lowest values for the d*y* displacement, 0.41 Å for EP and 0.39 Å for MO, while d*x* is 3.20 Å for EP and 2.67 Å for MO (Figure 3). A short d*y* displacement puts the two monomers nearly on top of each other, which combined with a large d*x* displacement places the ethyl substituent of one monomer close to the backbone of the second monomer (Figure 7 a). Analyzing the structure, one can observe interactions between the H atoms of the ethyl group and the C atoms that are involved in the active orbitals of the *S*_1_ and *T*_1_ states. These contacts are also present in the real EP structure but are missing when either an H or a methyl group is used to model the *R*_1_ substituent (see Figure 7, parts b and c). These interactions are stronger for the EP conformation, which shows the largest d*x* displacement and, consequently, the largest variation on the electronic coupling; somewhat smaller for the MO conformation, with similar d*y* but smaller d*x*, thus diminishing the overlap region of the ethyl of one monomer with the backbone carbon active orbitals of the second monomer. This hydrogen-backbone interaction is only of relative importance for the C8 conformation, with a similar d*x* displacement as the EP dimer but much larger d*y* slip-stacking displacement. For the remaining conformations, either with shorter d*x* displacement (see Figure 7d) or with larger lateral d*y* displacement (see Figure 7e), no significant intermonomer contacts are found, and the systems can be adequately modeled by an H atom instead of an alkyl group, as corroborated by the electronic couplings reported on Tables 4 and 7. Hence, for conformations with large d*x* and short d*y* displacements, due to the interactions of the substituents of one monomer with the carbon backbone of the neighboring monomer, the simplification of the substituent by an H atom is not the most appropriate one to evaluate electronic couplings.

**Table 7:** Direct and Total Electronic Couplings (in meV) between ^1^*TT* and *S*_0_*S*_1_ – *S*_1_*S*_0_ for the PDI Dimers for Which the Crystal Structure Is Available with *R*_1_ = Ethyl

	NOCI(x) direct	NOCI(x) total				
	x = CASSCF	x = CASSCF	x = CASPT2-0.25	x = CASPT2-0	x = SC-NEVPT2	x = PC-NEVPT2
EP	5.56	55.42	49.68	32.89	64.92	64.71
MO	4.48	42.42	39.91	23.18	49.53	49.35
C1	4.05	61.46	62.33	24.46	68.40	67.96
C3–I	2.12	43.50	54.22	17.13	53.17	51.78
C3–II	2.24	20.72	4.48	5.71	5.86	7.20
C7	5.62	73.66	88.21	26.45	87.30	85.29
C8	10.27	97.66	99.69	48.97	110.88	109.72

Heat maps of the electronic couplings for the scanned *x* and *y* slip-stacking displacements of the dimer computed with *R*_1_ = ethyl are reported in Figure S3 of Supporting Information. Since the effect of substituting the *R*_1_ group only influenced some conformations, the heat maps only slightly changed when replacing the H atom with an ethyl. Two regions of high coupling are clearly defined, one at intermediate d*x* and d*y* displacements and a small area at large d*x* and intermediate d*y* positions. Likely, ^1^*TT* formation rates, which depend on the value of the electronic coupling, are also affected when the H is substituted at *R*_1_ by an ethyl. The corresponding heat maps are presented in Figure S4 of Supporting Information, displaying also only slight changes. Again, the EP conformation emerges as the most favorable arrangement for singlet fission, followed by the MO conformation, and C32 as the less favorable geometrical disposition.

Overall, the *R*_1_ substituent chosen to model the PDI derivatives, does not significantly affect properties like the energies of the states of the monomer, the energies of the NOCI states in the dimers and the *E*_*SF*_ energies, however, for some conformations, it has a quantitative influence in the electronic couplings, which is a much smaller magnitude. This is an important finding since it is common practice to simplify the *R*_1_ substituent by an H atom to model chromophores and their derivatives uniquely on the basis of relative energies of the monomers.

## 4 Conclusions

Singlet fission energies, intermolecular electronic couplings, and relative ^1^*TT* formation rates have been computed by nonorthogonal configuration interaction (NOCI) calculations in a series of PDI dimers. The NOCI wave functions are written in terms of many-electron basis functions associated with physically sound electronic distributions in the dimers. This approach accounts for orbital relaxation and nondynamical electron correlation effects, while the remaining dynamical electron correlation has been introduced by a second-order perturbation theory energy correction. Inclusion of dynamical electron correlation effects is essential to properly describe the relative energies of the states involved in the SF process.

Results obtained for various PDI dimer conformations show that the ^1^*TT* state generated in the first step of the singlet fission process strongly mixes with other states, especially with charge transfer states. This mixing favors the electronic coupling between the initial *S*_0_*S*_1_ – *S*_1_*S*_0_ state and the ^1^*TT* state. NOCI(CASPT2-0) corrected wave functions show a tendency to overstabilize the ^1^*TT* state leading to a NOCI wave function with small contributions of charge transfer MEBFs, and consequently, the couplings computed including a CASPT2-0 electron correlation correction are systematically smaller than those obtained by NOCI(CASPT2-0.25) and NOCI(NEVPT2), where the ^1^*TT* state strongly mix with charge transfer states.

The geometrical conformations that best fulfill the thermodynamic criterion for singlet fission, i.e., being an exoergic or isoergic process, do not necessarily lead to larger electronic couplings. In fact, none of these criteria by itself can be taken as a unique reliable descriptor for optimal SF, instead, a combination of both relative energy and electronic coupling is important to establish the more favorable conformations for SF to occur. Computed relative rates for the ^1^*TT* formation points to dimer conformations with small displacements along the short axis and large shifts in the long axis as the most favorable region for the generation of the ^1^*TT* state. EP and MO PDI derivatives turn out to be the most suitable PDI compounds for SF. However, estimates of the ^1^*TT* binding energy based on the quintet state approximation reveal that the separation into two triplets is energetically unfavorable, thus inhibiting carrier diffusion. A more complete description of the triplet separation can be obtained by adding an extra monomer to the model and comparing the energy of the *T*_1_*T*_1_*S*_0_ state to the *T*_1_*S*_0_*T*_1_ state. Moreover, the NOCI calculation on the trimer provides the electronic coupling between the two states. This is subject of ongoing research.

The substituent at the imide functional group does not appreciably influence the relative energies of the different states; however, it has a significant effect on the values of the electronic coupling for some conformations where interactions between the substituent of one monomer and the carbon backbone of the nearest monomer come into play. This is the case for the EP and MO PDI derivatives and, to a lesser extent, the C8 PDI compound. Hence, the approximation of replacing the substituent with a H atom seems to be more appropriate to compute energies than to evaluate the electronic coupling.

## Supporting Information Available

Additional figures (Figures S1–S4) containing a representation of the active orbitals of the PDI monomer and the heat maps for the PDI dimer with an ethyl substituent and Tables S1–S4, including relative energies and wave function that support the findings of this study (PDF)
