# Supplementary material for: A Nonorthogonal Configuration Interaction Approach to Singlet Fission in Perylenediimide Compounds
Source: J Phys Chem A. 2023 Nov 15;127(47):9944–58. doi: 10.1021/acs.jpca.3c04975 (PMC10694806; doi:10.1021/acs.jpca.3c04975)
Supplement: Supplementary file 1 — jp3c04975_si_001.pdf [file jp3c04975_si_001.pdf]

Supporting information: A Non-orthogonal Configuration Interaction approach to Singlet Fission in Perylenediimide compounds

C. Sousa,[*,†] A. Sánchez-Mansilla,[‡] R. Broer,[¶] T. P. Straatsma,[§,‖] and C. de Graaf[‡,⊥]

†*Departament de Ciència de Materials i Química Física and Institut de Química Teòrica i Computacional, Universitat de Barcelona, 08028 Barcelona, Spain*

‡*Departament de Química Física i Inorgànica, Universitat Rovira i Virgili, 43007 Tarragona, Spain*

¶*Zernike Institute of Advanced Materials, University of Groningen, 9747 AG Groningen, The Netherlands*

§*National Center for Computational Sciences, Oak Ridge National Laboratory, Oak Ridge, TN 37831-6373, U. S. A.*

‖*Department of Chemistry and Biochemistry, University of Alabama, Tuscaloosa, AL 35487-0336, U. S. A.*

⊥*ICREA, Pg. Lluís Companys 23, 08010 Barcelona, Spain*

E-mail: c.sousa@ub.edu

Table S1: Relative energies (in eV) respect to the S_0 state for the PDI monomer with R_1=H calculated by various DFT methods. Experimental values[1,2] are also reported.

	S_1	T_1	$E(S_1)$- 2 $E(T_1)$
PBE-D3	2.41	1.28	-0.16
BP86-D3	2.05	1.27	-0.50
B3LYP-D3	2.63	1.45	-0.27
PBE0-D3	2.70	1.47	-0.23
ωB97X-D3	3.14	1.84	-0.54
CAM-B3LYP	2.98	1.69	-0.41
Experiment	2.34	1.19	-0.04

Table S2: Relative energies (in eV) respect to the S_0 state for the PDI monomer with R_1=H calculated by CASSCF(8,8), CASPT2-0.25 and CASPT2-0 applying four different basis set. Basis 1: ANO-RCC (5s,4p,2d,1f) contraction for C, N and O and (3s,2p,1d) for H. Basis 2: ANO-RCC (4s,3p,1d) contraction for C, N and O and (3s,1p) for H. Basis 3: ANO-RCC (4s,3p,1d) contraction for C, N and O and (2s) for H, and a def2-TZVP basis set. Experimental values are reported.[1,2]

	S_1	T_1	$E(S_1)$- 2 $E(T_1)$
Basis 1			
CASSCF(8,8)	4.25	2.28	-0.31
CASPT2-0.25	2.50	1.56	-0.62
CASPT2-0	1.69	0.75	+0.18
Basis 2			
CASSCF(8,8)	4.30	2.29	-0.29
CASPT2-0.25	2.58	1.59	-0.59
CASPT2-0	1.79	0.80	+0.19
Basis 3			
CASSCF(8,8)	4.32	2.30	-0.29
CASPT2-0.25	2.59	1.59	-0.60
CASPT2-0	1.81	0.81	+0.18
def2-TZVP			
CASSCF(8,8)	4.41	2.29	-0.17
CASPT2-0.25	2.48	1.57	-0.66
CASPT2-0	1.77	0.77	+0.23
Experiment	2.34	1.19	-0.04

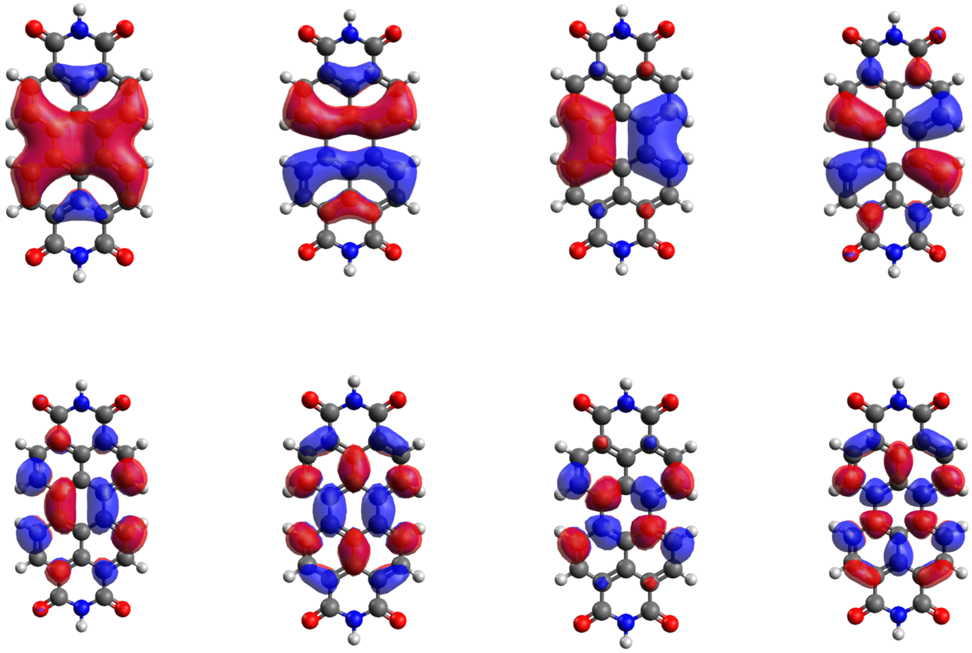

Figure S1: Active orbitals of the S_0 state of the PDI monomer with R_1=H. Active orbitals of the S_1, T_1, D^+ and D^- states are similar.

The fragment wave functions of all electronic states are computed by state specific CASSCF calculations under the spatial symmetry group of the monomer (D_{2h} or C_{2v}, depending on R_1). In the present case, the spatial symmetry of all PDI monomers considered is such that the S_0 and S_1 transform under different irreducible representations. The T_1, D^+ and D^- fragment states are also the lowest roots of a given irreducible representation. Hence, the optimization of the monomer wave functions does not suffer from root flipping and are easily identified. Afterwards, the fragment molecular orbitals are transformed to C_1 symmetry group to construct the MEBFs of the dimers.

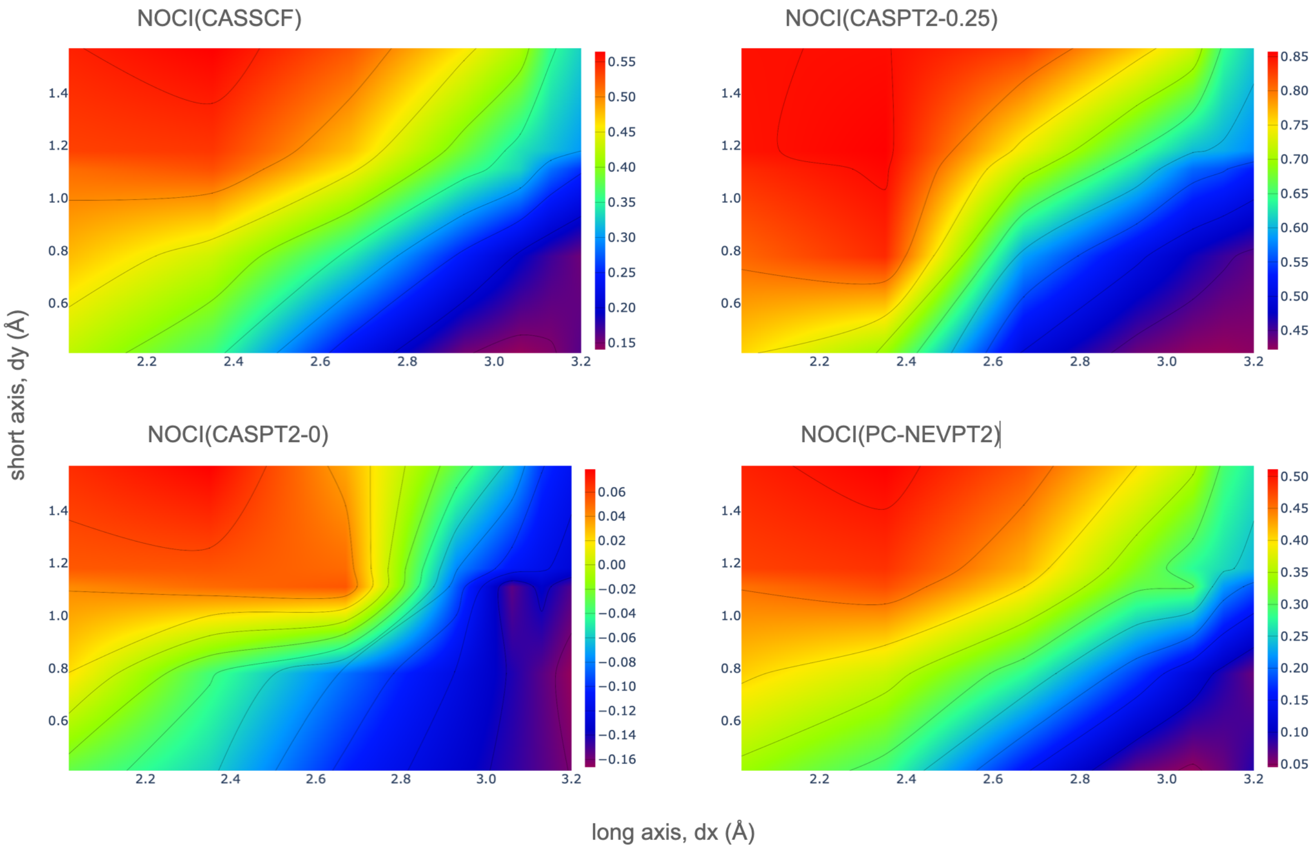

Figure S2: Singlet fission energy (eV), E_{SF}, computed by NOCI calculations, as funtion of the dx and dy displacements for PDI dimers with R_1=ethyl.

Table S3: MEBF coefficients and relative energies (eV) of the six NOCI wave functions for a PDI dimer at the C7 conformation with R_1=H. In bold coefficients larger than 0.15.

MEBF	Ψ_1	Ψ_2	Ψ_3	Ψ_4	Ψ_5	Ψ_6
NOCI(CASSCF)						
ΔE	0.00	4.26	4.48	4.71	4.85	4.92
S_0S_0	**0.999**	0.012	-0.003	0.024	0.001	-0.036
S_0S_1	0.019	**0.656**	**0.641**	**0.180**	**-0.296**	**-0.196**
S_1S_0	-0.020	**-0.654**	**0.643**	**-0.180**	**-0.296**	**0.197**
^1TT	0.001	-0.077	0.000	**0.849**	0.001	**0.522**
D^+D^-	-0.015	**0.246**	**0.283**	**-0.324**	**0.649**	**0.578**
D^-D^+	0.015	**-0.245**	**0.284**	**0.323**	**0.647**	**-0.580**
NOCI(CASPT2-0.25)						
ΔE	0.00	2.56	2.79	3.24	3.31	3.43
S_0S_0	**0.998**	0.029	-0.004	0.045	0.001	-0.038
S_0S_1	-0.030	**0.678**	**0.677**	**0.166**	**-0.201**	-0.116
S_1S_0	0.032	**-0.676**	**0.679**	**-0.166**	**-0.202**	0.117
^1TT	-0.001	-0.038	0.000	**0.676**	0.002	**0.736**
D^+D^-	0.024	**0.186**	**0.188**	**-0.492**	**0.681**	**0.473**
D^-D^+	-0.024	**-0.186**	**0.189**	**0.490**	**0.682**	**-0.474**
NOCI(CASPT2-0)						
ΔE	0.00	1.75	1.81	2.01	2.60	2.63
S_0S_0	**0.997**	-0.017	0.043	-0.005	0.001	-0.071
S_0S_1	-0.042	**-0.380**	**0.570**	**0.683**	**-0.179**	**-0.177**
S_1S_0	0.046	**0.378**	**-0.567**	**0.686**	**-0.177**	**0.179**
^1TT	-0.003	**0.808**	**0.577**	-0.001	0.001	0.120
D^+D^-	0.031	**-0.156**	0.092	**0.164**	**0.691**	**0.680**
D^-D^+	-0.031	**0.156**	-0.092	**0.166**	**0.684**	**-0.686**
NOCI(PC-NEVPT2)						
ΔE	0.00	1.73	1.94	2.08	2.23	2.28
S_0S_0	**0.996**	0.019	-0.004	0.049	0.003	-0.075
S_0S_1	-0.041	**0.614**	**0.561**	**0.239**	**-0.431**	**-0.253**
S_1S_0	0.045	**-0.612**	**0.562**	**-0.240**	**-0.429**	**0.259**
^1TT	-0.003	-0.128	0.001	**0.859**	0.004	**0.496**
D^+D^-	0.038	**0.326**	**0.417**	**-0.270**	**0.573**	**0.564**
D^-D^+	-0.038	**-0.325**	**0.421**	**0.266**	**0.567**	**-0.570**

Table S4: Relative energies (eV) of the six NOCI wave functions for the EP dimer with R_1: H, methyl, ethyl and ethylphenyl.

	Ψ_1	Ψ_2	Ψ_3	Ψ_4	Ψ_5	Ψ_6
NOCI(CASSCF)						
R_1=H	0.00	4.26	4.35	4.52	5.10	5.12
R_1=methyl	0.00	4.23	4.34	4.48	5.05	5.07
R_1=ethyl	0.00	4.31	4.39	4.56	5.12	5.14
R_1=ethylphenyl	0.00	4.32	4.37	4.56	5.15	5.17
NOCI(CASPT2-0.25)						
R_1=H	0.00	2.61	2.65	3.07	3.56	3.65
R_1=methyl	0.00	2.59	2.63	3.05	3.51	3.62
R_1=ethyl	0.00	2.66	2.69	3.12	3.58	3.69
R_1=ethylphenyl	0.00	2.65	2.66	3.12	3.60	3.70
NOCI(CASPT2-0)						
R_1=H	0.00	1.71	1.85	1.88	2.76	2.84
R_1=methyl	0.00	1.71	1.83	1.86	2.70	2.79
R_1=ethyl	0.00	1.76	1.90	1.92	2.79	2.86
R_1=ethylphenyl	0.00	1.73	1.88	1.88	2.82	2.88
NOCI(SC-NEVPT2)						
R_1=H	0.00	1.85	2.00	2.05	2.62	2.67
R_1=methyl	0.00	1.93	2.09	2.15	2.70	2.74
R_1=ethyl	0.00	1.91	2.03	2.13	2.67	2.70
R_1=ethylphenyl	0.00	1.90	2.00	2.11	2.68	2.71
NOCI(PC-NEVPT2)						
R_1=H	0.00	1.73	1.88	1.92	2.49	2.55
R_1=methyl	0.00	1.70	1.86	1.92	2.47	2.51
R_1=ethyl	0.00	1.78	1.91	2.00	2.55	2.58
R_1=ethylphenyl	0.00	1.78	1.88	1.98	2.56	2.59

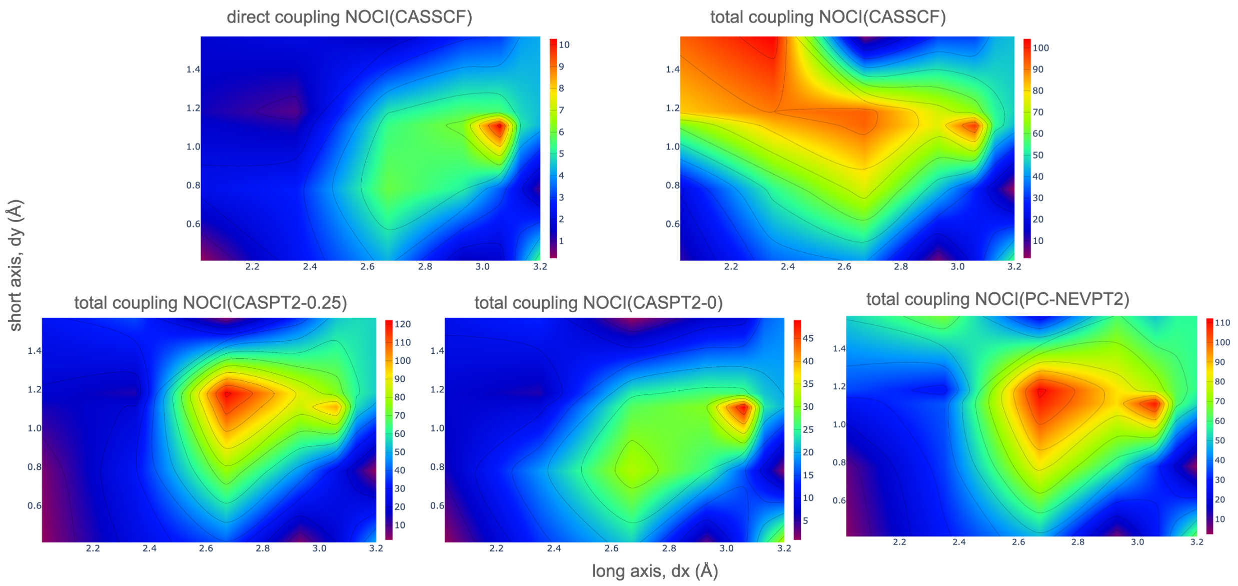

Figure S3: Electronic coupling (meV) between the ${}^1TT$ and $S_0S_1-S_1S_0$ states, computed by NOCI calculations, as function of the dx and dy displacements between two PDI molecules with R_1=ethyl.

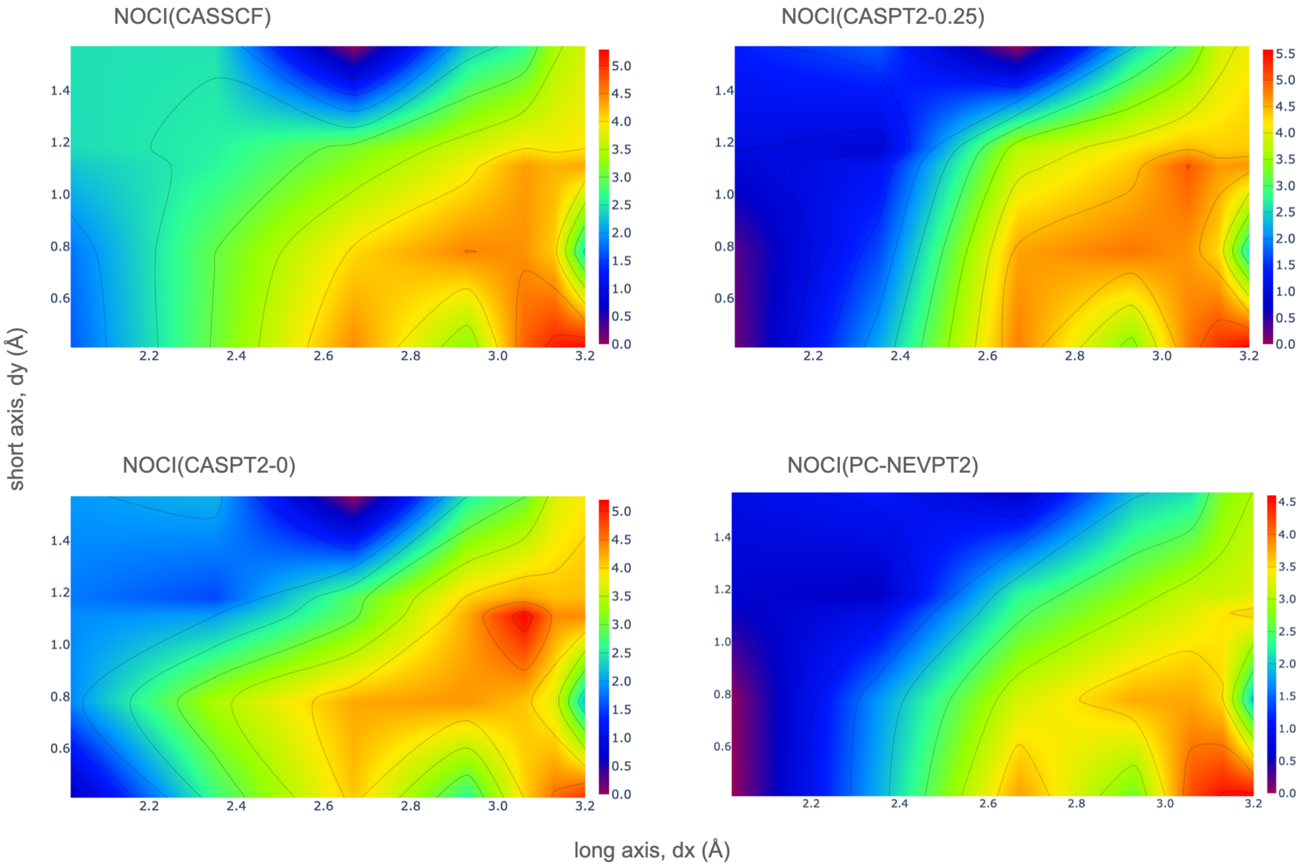

Figure S4: log of the relative singlet fission rates, computed by NOCI calculations, as function of the dx and dy displacement for PDI dimers with R_1=ethyl.

References

(1) Ford, W. E.; Kamat, P. V. Photochemistry of 3,4,9,10-Perylenetetracarboxylic Dlanhydrlde Dyes. 3. Singlet and Triplet Excited-State Properties of the Bis(2,5-di-tert-butylphenyl)imide Derivative. *J. Phys. Chem.* **1987**, *91*, 6373–6380.

(2) Le, A. K.; Bender, J. A.; Arias, D. H.; Cotton, D. E.; Johnson, J. C.; Roberts, S. T. Singlet Fission Involves an Interplay between Energetic Driving Force and Electronic Coupling in Perylenediimide Films. *J. Am. Chem. Soc.* **2018**, *140*, 814–826.
